# The Path to Fully Representational Theory of Mind: Conceptual, Executive, and Pragmatic Challenges

**DOI:** 10.3389/fpsyg.2020.581117

**Published:** 2020-11-04

**Authors:** Annelise Pesch, Andrei D. Semenov, Stephanie M. Carlson

**Affiliations:** Institute of Child Development, University of Minnesota, Saint Paul, MN, United States

**Keywords:** theory of mind, executive function, working memory, cognitive development, false belief

## Abstract

Although an explicit Theory of Mind (ToM) has been found to develop around 4 years of age in Western societies, recent work showing that 4- and 5-year-olds fail modified versions of False Belief tasks as well as seemingly easier True Belief tasks calls into question the robustness of preschoolers’ belief understanding. Some have argued these findings illustrate children’s conceptual limitations in their understanding of belief that are masked by standard False Belief tasks. However, others claim these examples of children’s failure can be explained by pragmatics of the testing situation, rather than conceptual limitations. Given the documented relation between ToM and executive function, an unexamined possibility is that children’s failure can be explained by certain executive demands. In the current study, we examined the relation between typically developing 4- (*n* = 43) and 5-year-olds’ (*n* = 42) performance on traditional and modified False Belief tasks, True Belief tasks, and one component of executive functioning - working memory. We found that children performed worse on modified False Belief tasks and True Belief tasks compared to standard 2-option False Belief tasks, and that working memory was related to modified 3-option contents False Belief performance. These results suggest that a fully representational ToM, one that is stable in the context of increased conceptual, executive, and pragmatic demands, may develop later than traditional accounts have assumed.

## Introduction

Theory of Mind (ToM) is a social cognitive skill that refers to the ability to understand and reason about other people’s mental states, including beliefs. Achieving ToM understanding allows children to succeed in social environments, such as school, and therefore understanding the developmental timeline of ToM is informative to various intervention programs and curricula (*for review*, Carlson et al., 2013). A representational ToM refers to the view that beliefs and desires are representations of the real world and that these representations mediate our actions in the world. Our beliefs about the world can be either true or false and our intentions and desires can be either fulfilled or unfulfilled. We act to fulfill our desires in light of our beliefs and therefore if we know somebody’s beliefs and desires we can predict how they will act in a certain situation (Perner, 1991). According to this view, if somebody performs a misguided action then this is either because they have a false belief or an unaligned desire.

However, the traditional tools used to measure ToM understanding may not be telling the whole story. Indeed, facets of the tasks such as how the scenario is presented or whether there are additional attentional demands on the child may change how a child responds to a ToM task. Therefore, understanding the underlying demands of traditional ToM tasks can help researchers better trace the development of ToM skills and provide insight into future intervention programs.

### Standard Theory of Mind Measures

Traditionally, children’s attainment of ToM is measured by a False Belief task in which a child must answer in accordance with what a character believes, even if that belief contradicts the reality of the situation (Wimmer and Perner, 1983). In the False Belief Contents task (Hogrefe et al., 1986), children witness a container (e.g., an M&M box) and are shown that it contains an unexpected object (e.g., key). The child is asked what someone else, who has never seen inside this box before, would think is in the container. If children are reasoning about another person’s belief, they have to ignore the reality (that the key is in the box) and respond that another person would think M&Ms are in the M&M container. Similarly, in the Location variant of the task (Wimmer and Perner, 1983), children witness a scenario in which a protagonist places an object into one of two locations (Location A), then the protagonist leaves the room and another character moves the object to another location (Location B) and the child is asked where will the protagonist look for the object upon returning to the room. For children to reason about belief, they have to ignore the reality that the object is in Location B and say that the character would look for the object in Location A. Although performance on False Belief tasks can vary by age depending on the type of questions being asked and the scenarios presented (Wellman and Liu, 2004), typically, 4 to 4.5-year-olds pass the standard False Belief task, whereas younger children fail (Wellman et al., 2001). It should be noted, however, that there is evidence to suggest false belief understanding and its precursors as early as infancy using other dependent measures such as eye gaze (*for review*, Clements and Perner, 1994; Carlson et al., 2013).

### Role of Executive Function

Performance on ToM tasks is robustly linked with individual differences in children’s executive function (EF) skills (for a meta-analysis, see Devine and Hughes, 2014). EF refers to neurocognitive skills involved in goal-directed control of behavior and thoughts; these skills include inhibitory control, cognitive flexibility and working memory (Miyake et al., 2000; Zelazo et al., 2016). According to one account of these results, EF skills allow children to *express* their knowledge of ToM. For example, a 3-year-old child might be reasoning about a character’s belief that the object is in Location A, but the most recent move to Location B created a strong representation that they are unable to inhibit. Over time, as inhibitory control develops, children can more accurately express their existing ToM knowledge (Carlson et al., 1998). Alternatively, EF skills might make it possible for children to first suppress their own salient thoughts and beliefs, a *sine qua non-for* reasoning about the beliefs of others. On this account, EF skills facilitate the *emergence* of ToM (Carlson and Moses, 2001; Moses, 2001).

### Perceptual Access Reasoning

Both expression and emergence accounts highlight the contributions of EF skills to ToM, but they rely on successes and failures on the standard False Belief task, a task whose utility has come into question as being the primary indicator of explicit ToM attainment (Fabricius et al., 2010). A critique of this task suggests that passing the False Belief task can be achieved by reasoning about a protagonists’ perceptual access to a set of events, rather than the protagonists’ beliefs (Fabricius and Khalil, 2003). According to the Perceptual Access Reasoning (PAR) hypothesis, children reason that agents with perceptual access have knowledge, whereas agents who lack perceptual access do not have knowledge. Crucially, this type of reasoning process does not involve attributions of mental states (intentions, desires, and beliefs) to agents. For example, if a protagonist places an object in Location A and then someone moves the object to Location B while the protagonist is watching, then the child reasons that the protagonist did not lose perceptual access, knows where the object is, and will therefore search correctly at Location B. However, if the protagonist was absent from the room when the object was moved, then the child reasons that the protagonist does not know where the object is and will search incorrectly at Location A, not because the child is drawing on a representational understanding of mental states, but because the protagonist’s perceptual access to the event was broken. This reasoning strategy results in passing standard 2-option False Belief tasks. According to the PAR hypothesis, the traditional 2-option False Belief task is limited in terms of disentangling the traditional perspective on ToM and the PAR account because the incorrect choice and the belief choice are one and the same.

Support for the PAR account emerges from two primary findings. The first piece of evidence for the PAR account comes from a modified False Belief task that includes an additional incorrect response option, thereby disambiguating a PAR generic incorrect response from a traditional ToM reality-based incorrect response (Fabricius and Khalil, 2003). In the Location task, Location C is added such that it is present in the room alongside Location A and Location B, but is neither the original hiding location (belief response) nor final hiding place (reality response). According to the PAR hypothesis, a child who witnesses a protagonist lose the chain of perceptual access will reason the protagonist will not know where the object is and will thus be wrong when searching for the object. Given the presence of two incorrect options (belief and irrelevant), the child should arbitrarily select one of them. Indeed, Fabricius and Khalil (2003) found that a modified 3-option False Belief task showed higher failure rates for 5-year-olds (65%) than on the traditional 2-option False Belief task (36%).

The second source of support for the PAR account involves children’s performance on True Belief tasks. True Belief tasks originally were used with 3-year-olds to demonstrate that False Belief failure is not accounted for by incidental task demands because they were able to pass the True Belief tasks but failed the False Belief tasks (Wellman et al., 2001). True Belief tasks are not typically administered to 4-year-olds because they are able to pass the presumably more difficult False Belief version. Structurally, True Belief and False Belief tasks are similar: the child observes as a protagonist hides an object in Location A, leaves the room, and another character moves the object to Location B. However, then the character moves the object *back* to Location A. The child is asked where the protagonist will look for the object upon returning to the room. In this task, the reality and the belief option are the same and moving the toy was inconsequential, so the child should say the protagonist will look in Location A. Yet, for 4-year-olds who typically pass the False Belief task, a large proportion of them fail the True Belief variant (Fabricius and Khalil, 2003; Fabricius et al., 2010). On the PAR account, children reason that seeing/not seeing → knowing/not knowing and that knowing/not knowing → getting it right/getting it wrong (Hedger and Fabricius, 2011). Therefore, they would expect the protagonist to search incorrectly (getting it wrong) on this task by virtue of having been absent during the hiding events (not seeing), even though the object is, in fact, right where they last saw it. Hence, the very same heuristic that led to an apparently correct response on the standard False Belief task would lead 4-year-olds to respond incorrectly on the True Belief task. Based on 4- and 5-year-olds’ performance on modified False Belief tasks and True Belief tasks, the PAR hypothesis argues that children do not attain a fully representational ToM until closer to age 6.

### Pragmatics

Although 4- and 5-year-olds’ failure on modified 3-option False Belief tasks and True Belief tasks supports the PAR hypothesis, other work suggests that performance may be influenced by pragmatic demands, which would preserve children’s conceptual understanding of belief. For example, a replication study conducted by Perner and Horn (2003) found that children performed well on both standard 2-option and modified 3-option False Belief tasks. The authors argued that the poor performance reported by Fabricius and Khalil (2003) could be attributed to the use of three yes-no test questions (which might confuse children), instead of an open ended test question. Similar arguments have been made in response to children’s counterintuitive True Belief performance. Oktay-Gür and Rakoczy (2017) argued that a sufficiently modified True Belief task in which the critical change of location occurs in the presence of the character prior to them leaving the room (and breaking their perceptual access) is associated with improved performances for 4- and 6-year olds. Rakoczy and Oktay-Gür (2020) systematically examined how the communicative pragmatics of True Belief tasks might lead 4-year-olds to fail whereas 3-year-olds pass. In particular, they found that when True Belief tasks were administered first and False Belief tasks second, performance on True Belief was much better than if the order was reversed. The authors interpreted these findings to suggest that the perceived ease of the True Belief questions made children think there was some trick or that the examiner wanted a non-obvious response when the question came after the False Belief question. Furthermore, if children were given context about the task, explaining that some questions were easier and were designed for younger kids, then performance on True Belief tasks also increased.

### Working Memory

Yet another explanation for the evidence concerning the modified False Belief task remains unexamined. Specifically, the inclusion of a third option might increase the strain on children’s working memory, making performance on the task lower than the traditional 2-option task. Similarly, when considering 2nd order False Belief tasks where a child must reason about another person’s false belief about the protagonist’s false belief, the added level increases the executive function demands and performance declines (Happé, 1994; Miller, 2009). If working memory demands are increased by adding a third option to the traditional task, then one would expect that performance will continue to decrease with additional options. Therefore, it is possible that pragmatic demands of the True Belief task being administered to older children, along with increased working memory demands placed by the modified 3-option task, suggest alternatives to be considered alongside the PAR hypothesis.

### Present Study

The present study sought to address the conceptual, pragmatic, and executive issues that constrain children’s performance on modified multi-option False Belief tasks. The *conceptual* account suggests that children do not yet have a fully representational ToM by 4 years of age and that their apparent success on the 2-option False Belief tasks is due to a confound in task design. The PAR hypothesis suggests the relatively poor performance on 3-option versions (where they are just as likely to choose the irrelevant response as the belief response) reveals that young children are using a simpler heuristic akin to, “Did the protagonist see the turn of events?” as opposed to representing the protagonist’s mental state of belief. Alternatively, the *executive* account explains differences between performance on 2- and 3-option False Belief tasks through the added demands on executive function, specifically working memory. Given the robust association between EF and ToM (Devine and Hughes, 2014), it might be the case that additional options pose a challenge to children’s under-developed working memory capacity, thus impeding their ability to express their ToM.

To arbitrate these competing arguments, we tested the role of working memory in performance on multi-option False Belief tasks in multiple ways. First, we added a 4-option task to the 2- and 3-option versions. This addition allowed us to test for the contributions of working memory to modified False Belief task performance, such that performance on the 4-option task would be poorer than the 3-option task which in turn is poorer than the 2-option task. The second way we examined this issue was to administer independent tests of working memory to explore associations between working memory and ToM performance. Third, we tested for pragmatics with our use of open-ended test questions in standard and modified False Belief tasks and inclusion of task order in our analyses. On the PAR hypothesis, 4- and 5-year-olds’ performance should reflect patterns reported in prior investigations. Specifically, children should pass standard 2-option False Belief tasks but fail the True Belief tasks and modified 3- and 4-option False Belief tasks, and this pattern should not be associated with working memory. On the other hand, if working memory is associated with performance on multi-option False Belief tasks or True Belief tasks, then this would offer support for an executive account. Finally, support for a pragmatics account would be reflected by children passing standard and modified 3-option False Belief tasks (due to the use of an open-ended test question), as well as a significant effect of task order on True Belief task performance.

Next, our study was positioned to address disparate findings between the Contents and Location variants of the ToM tasks. In particular, studies have found that 4-year-olds perform worse on the Contents variant than the Locations variant (Fabricius et al., under review; Fabricius and Khalil, 2003; Perner and Horn, 2003). It is possible that these findings could be explained by differing working memory demands inherent in Contents or Location variants. In the Contents task, greater working memory may be required to hold in mind the various contents and select the correct response among them.

Finally, our study presented an opportunity to explore the anomalous findings of 4-year-old children failing True Belief tasks while passing False Belief tasks. The *pragmatics limitation* account suggests that there are aspects of task administration that make it difficult for older children to pass the True Belief tasks, specifically due to the pragmatics of the task. For instance, presenting such an “easy” question to a child might make them confused and second guess their answer, especially if it followed a false belief question (Oktay-Gür and Rakoczy, 2017; Rakoczy and Oktay-Gür, 2020).

## Materials and Methods

### Participants

Eighty-five children participated in the study [47 female, *M*_age(months)_ = 60.50; *SD* = 7.00, range = 49.60–71.80 months], including 43 4-year-olds [28 female, *M*_age(months)_ = 54.45; *SD* = 2.96, range = 49.60–59.40 months] and 42 5-year-olds [19 female, *M*_age(months)_ = 66.68; *SD* = 3.73, range = 60.60–71.80 months]. This sample size was based on having 80% power to detect a moderate effect size of *f* = 0.36. Five participants were excluded from analyses, due to examiner error (*n* = 2), child refusal (*n* = 2), and one child was discovered to be the twin of a previous participant after data were already collected. Participants were selected from a university-maintained database of children living near a large Midwestern city. Children from this database are primarily White, native English speakers from middle to high socioeconomic status (SES) households. Upon concluding the visit, children selected a plastic toy prize (valued < $1) and were given a lab T-shirt. Parents were also given a $10 gift card.

### Procedure

All children were tested individually in a single 30-min videotaped session by one of two graduate research assistants. The measures included a ToM battery and a working memory battery. Tasks were administered in three blocks, each consisting of two ToM tasks (a Contents and a Location variant) followed by a working memory task. Task order was counterbalanced using a Latin square design that preserved ToM tasks of the same number of options (e.g., 3-option False Belief task) within the same block while counterbalancing the order in which the blocks and the working memory tasks appeared. As a result, there were 8 task orders, 2 task orders presented the 2-option True Belief tasks first (*n* = 20) and 6 task orders presented some variant of the False Belief task first (*n* = 65).

### Measures

#### Working Memory Measures

##### Corsi Blocks (Corsi, 1972)

Children were asked to point to a series of wooden blocks arranged on a physical board in an irregular order. The first block of trials, forward span, required children to repeat a pattern of tapping blocks exactly as E demonstrated. Children started with a practice span of 1 and then 2 taps and then continued to test spans of 2 blocks up to a potential span of 9 blocks. If a child failed a certain span length then they would be administered an additional pattern at the same span length. If a child failed two patterns at the same span length then the administration concluded. After the forward span block, children proceeded to the backward span block where children they were required to tap blocks in the reverse order as E. As with the forward span block, children who failed on a given pattern were given one more pattern at the same span, and two failed patterns of a given span concluded the task.

##### Word Span (Carlson et al., 2002)

Children were asked to repeat a list of words back to E (forward span) and in reverse order (backward span). The forward span block was always given before the backward span block. Children were introduced to the task with a puppet (Ernie) who demonstrated saying words forward (e.g., E said “*bear*, *hat*” and Ernie replied “*bear*, *hat*”) or backward (e.g., E said “*book*, *cup*” and Ernie replied “*cup*, *book*”). Children received a practice trial for each span direction and were corrected if necessary. Test trials started with a span length of 2 and increased to a max span of 5. If children correctly repeated the words without errors, then E would proceed to next span length. If a child failed at a given span length, they would then be given up to two more word sets at the same span length before terminating the task.

##### Count and Label (Gordon and Olson, 1998)

In this measure of dual-task performance, children were asked to count and label objects presented to them. E presented the child with three objects (key, comb, and toy dog), naming and pointing to each. Next, E counted as they pointed to each object (one, two, and three). Finally, E pointed, counted, and named each object in turn: “*One is a key*, *two is a comb*, *three is a dog.*” Children were given their own set of items (doll, shoe, and block) and were asked to repeat the steps E took (first label the items, then count the items, then count and label the items). Children repeated the counting and labeling of the same items twice and scores were given for the number of trials (out of two) they completed correctly.

#### Theory of Mind Measures

##### True Belief

There were two True Belief tasks, a Contents version and a Location version. The tasks were modeled after previous work investigating the PAR hypothesis (Fabricius et al., 2010).

###### Contents

In the True Belief Contents task, children were shown an M&Ms candy box and asked what they thought was inside. Children were corrected with a series of prompts if they did not state M&Ms or candy (e.g., *“What kinds of things come in a box like this?”*). Children were then shown the contents of the box (a key) and allowed to touch it before E placed it next to the box on the table. E then produced a cup filled with M&Ms and poured into the box while stating, “*Here*, *let’s put some candy inside.*” Children were then asked two control questions: “*What is inside the box now?”* and “*What was inside the box when I first showed it to you?”* Incorrect responses were corrected and re-asked. The empty cup and key were then removed from the table. E asked the test question, *“Let’s pretend I have a friend named John waiting right outside the door. He’s never seen inside this box. When he first looks at the box*, *before he opens it*, *will he think there is a candy or a key* [counterbalanced] *inside?”* Children were then asked an open ended justification question, *“Why will he think there is a candy/key inside?”*

###### Location

In the True Belief Location task, a red and blue box were placed on the table and children were introduced to Sarah, who wanted to save her toy for later. Sarah placed her toy in the red box and then sat in between the two boxes. Sarah’s dad entered and was described as cleaning Sarah’s room. Dad moved the toy from the red box to the blue box, stating *“Watch Sarah*, *I’m moving your toy.”* Then Sarah and her dad were removed from the table. Children were asked three control questions: *“Remember when Sarah was here*, *where did Sarah put the toy away?”*, *“Did Sarah watch him move her toy?”*, and *“Where did Sarah’s dad move the toy to?”* Children who failed a control question were retold the story and the question was repeated. Children were then asked the test question, *“Look*, *Sarah comes back to get her toy and stands right here* [between the cupboards], *where does she think her toy is?”*

##### False Belief

The False Belief battery included standard and modified versions of Contents and Location tasks. There were 6 tasks: two 2-option False Belief (standard Contents and Location), two 3-option False Belief (modified Contents and Location), and two 4-option False Belief (modified Contents and Location). The modified 3- and 4-option False Belief tasks were modeled after prior work investigating the PAR hypothesis (Fabricius and Khalil, 2003; Fabricius et al., under review). Responses to standard versions of the task included two options: reality and belief. Responses to modified versions of the task included three options: reality, irrelevant, and belief. The tasks are described in detail below.

###### Contents

In all three versions (2-option, 3-option, and 4-option) of the False Belief Contents task, children were shown a familiar box (Crayon box, Band-Aid box, or Cookie box) and asked what they thought was inside. Children were corrected with a series of prompts if they did not state the contents displayed on the box (e.g., “*What kinds of things come in a box like this?”*). Children were then shown the contents of the box and depending on the version of the task, a series of objects were revealed and placed back in the box.

In the 2-option version, a pencil [reality] was removed from the box and then placed back inside. Children were asked two control questions: *“What kind of box is this?”* and *“What is inside the box now?”* E asked the test question: *“Let’s pretend I have a friend named Sam waiting right outside the door. He’s never seen inside this box. When he first looks at the box*, *before he opens it*, *will he think there is a pencil or crayons* [counterbalanced] *inside?”* Children were then asked an open-ended justification question, *“Why will he think there is a pencil/crayons inside?”*

In the 3-option version, a toy car [irrelevant] was removed from the Band-Aid box. E then produced a spoon [reality] and placed the spoon inside the box. Children were asked two control questions: *“What was in the Band-Aid box in the beginning?”* and *“What is in the box now?”* E removed the toy car from the table, produced a toy doll, and asked the test question (*“Here comes Kate. Kate has never seen inside this box. What does Kate think is in the box?”*) and the memory control question (*“Did Kate see inside this box?”*).

In the 4-option version, a coin [irrelevant] was removed from the Cookie box. E then produced a rock [irrelevant] and placed the rock [irrelevant] inside the Cookie box. E then produced a block [reality]. E removed the rock from the box and replaced it with the block. Children were asked three control questions: *“What was in the Cookie box in the beginning?”*, *“What did we put in the box next?”*, and *“What is in the box now?”* E removed the coin and rock from the table, produced a toy doll, and asked the test question (*“Here comes Mark. Mark has never seen inside this box. What does Mark think is in the box?”*) and the memory control question (*“Did Mark see inside this box?”*).

###### Location

In all three versions (2-option, 3-option, and 4-option) of the False Belief Location task, boxes were produced and children were told a story about a set of characters.

In the 2-option version, a green and white box were placed on the table and children were introduced to Spot, a dog who wanted to save his favorite treat for later. Spot placed his treat in the white box [belief] and then went outside to play. Spot’s friend Fluffy the cat entered and moved the treat to the green box [reality] and then left as well. Children were asked four control questions: *“Where is the treat now? [reality]”*, *“Where was the treat in the beginning? [belief]”*, *“Who moved it to the green box?”*, and *“Could Spot see that?”* Children who failed a control question were retold the story and the question was repeated. Children were then asked the test question, *“Now Spot comes back to get his treat. Where will Spot first look for his treat?”*

In the 3-option version, a red, blue, and white box were placed on the table and children were introduced to Anna and her dad. Dad brought Anna a chocolate bar and, while she watched, placed it in the blue box [irrelevant]. Dad decided to move the chocolate from the blue box to the red box [belief]. Then Anna left the room, and Dad moved the chocolate to the white box [reality] and left as well. Children were asked four control questions: *“Where did Anna watch Dad put the chocolate first? [irrelevant]”*, *“Where did Anna watch Dad put the chocolate next? [belief]”*, *“Then Anna left*, *and where did Dad put it when she was gone? [reality]”*, and *“Did Anna see her dad move it to the white box?”* Children who failed a control question were retold the story and the question was repeated. Children were then asked the test question, *“When Anna comes back to get her chocolate*, *where will she first look for her chocolate?”*

In the 4-option version, red, white, blue, and green boxes were placed on the table and children were introduced to Sam and his mom. Mom brought Sam a chocolate bar and, while he watched, placed it in the blue box [irrelevant]. Mom decided to move the chocolate from the blue box to the white box [irrelevant]. Mom then decided to move the chocolate from the white box to the green box [belief]. Then Sam left the room, and Mom moved the chocolate to the red box [reality] and left as well. Children were asked five control questions: *“Where did Sam watch Mom put the chocolate first? [irrelevant]*,*” “Where did Sam watch Mom put the chocolate next? [irrelevant]*,*” “Where did Sam watch Mom put the chocolate after that? [belief]*,*” “Then Sam left*, *and where did Mom put it when she was gone? [reality]*,*”* and *“Did Sam see his mom move it to the red box?”* Children who failed a control question were retold the story and the question was repeated. Children were then asked the test question, *“When Sam comes back to get his chocolate*, *where will he first look for his chocolate?”*

## Results

### Working Memory Assessments

Children’s performance on working memory tasks can be seen in Table 1. As shown in Table 2, Both Corsi Block and Word Span tasks were correlated with each other, even after controlling for age, whereas Count and Label was correlated with Backward Word Span but not with Corsi Block or with age. Both 4-year-olds and 5-year-olds performed at ceiling for Count and Label. Given the ceiling effect and the lack of consistent correlations with other working memory measures, Count and Label task was excluded from further analyses. Thus, we created a Working Memory Composite by averaging *z*-scores of highest level passed on Backward Corsi and Backward Word Span. There were age-related differences in working memory such that 5-year-olds had higher working memory composites than 4-year-olds, *t*(71.24) = −3.85, *p* < 0.001. There were no differences in working memory related to gender.

**TABLE 1:** Working memory task performance by age group.

	4-year-olds			5-year-olds		
Task	Min	Max	Mean (*sd*)	Min	Max	Mean (*sd*)
Corsi block	1	4	2.35 (0*.95*)	1	5	3.19 (*1.15*)
Backward word span	1	3	2.05 (0*.87*)	1	4	2.50 (0*.80*)
Count and label	0	2	1.16 (0*.81*)	0	2	1.45 (0*.71*)

*Italicized values are indicated to be (SD)-standard deviation.*

**TABLE 2:** Correlations among study variables.

	1	2	3	4	5	6	7	8	9	10	11	12
(1) Backward Corsi Span		0.10	0.40***	0.83***	−0.20†	−0.12	0.01	−0.09	0.13	0.10	0.11	0.05
(2) Count and label	0.18		0.43***	0.32***	0.00	−0.01	0.01	−0.02	0.05	0.05	0.01	0.02
(3) Backward word span	0.47***	0.46***		0.85***	−0.20†	−0.19†	0.14	0.17	0.18†	0.14	0.13	−0.04
(4) WM composite	0.86***	0.37***	0.86***		−0.24*	−0.18	0.09	0.05	0.19†	0.15	0.15	0.00
(5) TB contents	−0.12	0.03	−0.15	−0.15		0.26*	−0.09	−0.14	−0.11	−0.11	−0.13	−0.16
(6) TB location	−0.09	0.00	−0.17	−0.16	0.26*		−0.19	−0.09†	−0.20	−0.10†	−0.33***	−0.06
(7) FB 2 contents	0.11	0.07	0.20†	0.18†	−0.05	−0.17		−0.20†	0.41***	0.15	0.26*	0.12
(8) FB 2 locations	−0.02	0.01	0.20†	0.11	−0.11	−0.08	−0.16		0.02	0.13	0.24*	0.21†
(9) FB 3 contents	0.28**	0.13	0.27*	0.33***	−0.04	−0.17	0.47***	0.08		0.31**	0.62***	0.25*
(10) FB 3 locations	0.24*	0.12	0.23*	0.28*	−0.05	−0.08	0.23*	0.17	0.41***		0.30*	0.42***
(11) FB 4 contents	0.21†	0.06	0.20†	0.24*	−0.09	−0.31**	0.31***	0.27*	0.65***	0.36***		0.18
(12) FB 4 locations	0.19†	0.09	0.06	0.14	−0.10	−0.05	0.20†	0.24*	0.36***	0.49***	0.25*	
(13) Age (months)	0.43***	0.21†	0.28**	0.41***	0.15	0.03	0.25*	0.14	0.40***	0.37***	0.26*	0.34***

Below diagonal are bivariate correlations. Above diagonal are partial correlations controlling for age. Computed correlation used Pearson method with pairwise deletion (FB, false belief; TB, true belief). *p < 0.05, **p < 0.01, ***p < 0.001, ^†^p < 0.10. Bold values indicate statistically significant correlations.

### True Belief Task Performance

Although the primary aim of this study was to examine the contributions of working memory to modified false belief task performance, we also examined children’s performance on 2-option True Belief tasks given recent work suggesting some children perform worse on such tasks compared to standard (2-option) False Belief tasks. We first examined the correlations between working memory and True Belief task performance. Inspection of the raw and partial correlations (controlling for age) revealed non-significant correlations (see Table 2).

**FIGURE 1:**
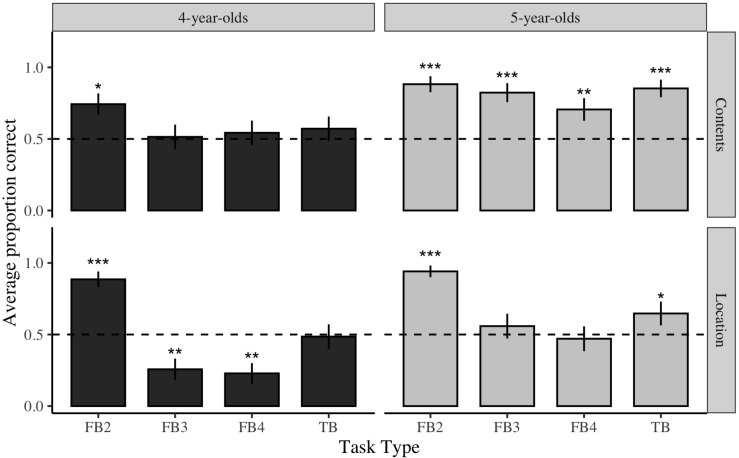
Average performance on each ToM tak as a function of task version and age. ^∗^*p* < 0.05, ^∗∗^*p* < 0.01, ^***^p < 0.001. Dashed line represents chance performance on task.

Next, we compared performance across True Belief and standard (2-option) False Belief tasks. A logistic mixed effects model was conducted to predict score (1: Pass, 0: Fail) from the fixed effects of task order, age (months), task type (True Belief vs. False Belief), task version (Contents vs. Location), the interaction between task type and task version, and the random effects (intercept) of participants. The analysis revealed a significant effect of age (б = 0.05, *p* = 0.01). There was also a significant effect of task version (б = 1.13, *p* = 0.01), with better performance on Location versions of the task compared to Contents versions. In addition, there was a significant interaction between task version and task type (б = −1.77, *p* = 0.002). While children’s performance on Contents versions of the True Belief and False Belief tasks was similar, they performed significantly better on the Location version of the False Belief task compared to the Location version of the True Belief task (see Figure 1). Task order was not significant.

Both age groups performed significantly above chance on Contents and Location versions of the standard 2-option False Belief tasks [4-year-olds Contents FB: *t*(42) = 2.41, *p* < 0.05; 4-year-olds Location FB: *t*(41) = 7.531, *p* < 0.0001; 5-year-olds Contents FB: *t*(41) = 7.53, *p* < 0.0001; 5-year-olds Location FB: *t*(40) = 13.25, *p* < 0.0001]. In contrast, on True Belief tasks, 5-year-olds performed significantly above chance on both versions of the task [5-year-old Contents TB: *t*(39) = 4.68, *p* < 0.0001; 5-year-old Location TB: *t*(39) = 1.96, *p* < 0.05], whereas 4-year-olds’ performance did not differ from chance on either version of the task [4-year-olds Contents TB: *t*(40) = 1.76, *p* = 0.08; 4-year-olds Location TB: *t*(42) = 0.15, *p* = 0.88].

### False Belief Task Performance

Children’s average performance across the set of control questions was uniformly high, ranging from 96 to 100% [True Belief Contents: 98%; True Belief Location: 99%; standard (2-option) False Belief Contents: 100%; standard (2-option) False Belief Location: 99%; 3-option False Belief Contents: 100%; 3-option False Belief Location: 99%; 4-option False Belief Contents: 99%; 4-option False Belief Location: 97%]. Children who answered a control question incorrectly were not given credit for passing.

Given recent work suggesting that pragmatic demands can impede children’s performance on true belief tasks (e.g., Rakoczy and Oktay-Gür, 2020), we included task order in all models described below. Task order indicates which ToM task children received *first*: Order 1: True Belief, Order 2: standard False Belief, Order 3: 3-option modified False Belief, Order 4: 4-option modified False Belief.

To begin, we compared performance across standard and modified tasks. To maintain consistency across the set of tasks, reality and irrelevant responses were both coded as 0 to indicate an incorrect response on modified 3- and 4-option False Belief tasks. A logistic mixed effects model was conducted to predict score (1: Pass, 0: Fail) from the fixed effects of task order, age (months), task type (True Belief, standard (2-option) False Belief, 3-option False Belief, 4-option False Belief), task version (Contents vs. Location), the interaction between task type and task version, and the random effects (intercept) of participants.

The analysis revealed a significant main effect of age, as well as several two-way interactions between task type and task version (see Table 3). To better understand the pattern of performance across the battery of ToM tasks, the proportional scores were compared with chance, as shown in Figure 1. Here, chance was defined as a 50/50 pass (providing the belief response) or fail (providing either the irrelevant or reality response). Inspection of Figure 1 reveals two things. First, whereas children across both age groups performed better on Location versions than Contents versions of standard (2-option) False Belief tasks, they performed better on Contents versions of modified 3- and 4-option False Belief tasks and True Belief tasks. Second, modified 3- and 4-option False Belief tasks were more difficult for children, especially for 4-year-olds compared to 5-year-olds. Specifically, whereas 4-year-olds’ performance on Contents and Location versions of standard (2-option) False Belief tasks was high, they were at or below chance on all other ToM assessments. Five-year-olds performed well across contents versions of both standard and modified False Belief tasks, but their performance dropped to chance on Location versions of the modified False Belief tasks.

**TABLE 3:** Results of mixed logistic regression model predicting odds of choosing belief by age, task order, task type, and task version.

Predictor	Odds ratios	Conf. Int (95%)	*P*-value
Intercept	0.03	0.00–0.31	0.003
Task order	0.79	0.62–1.01	0.062
Age (months)	1.10	1.06–1.14	**<0.001**
Task (modified 3-options FB)	0.42	0.18–0.97	**0.042**
Task (modified 4-option FB)	0.34	0.15–0.77	**0.010**
Task (true belief)	0.53	0.23–1.24	0.143
Task version (location)	2.69	0.92–7.87	0.071
Task (modified 3-option FB) * Version (location)	0.10	0.03–0.39	**0.001**
Task (modified 4-option FB) * Version (location)	0.10	0.03–0.36	**0.001**
Task (true belief) * Version (location)	0.18	0.05–0.66	**0.010**
**Random effects**			
∫^2^	3.29		
|00 subject	0.37		
ICC	0.10		
*N* subject	69		
Observations	552		
Marginal *R*^2^/conditional *R*^2^	0.303/0.373		

*The reference group for Task was 2-option FB. The reference group for Task Version was Contents. Bold values are statistically significant values.*

To better understand poor performance on the modified False Belief tasks, we examined the proportion of children choosing the reality versus irrelevant option, as these responses reflect different ways of “getting it wrong.” In 3-option False Belief tasks, choice of the third “irrelevant” option suggests children’s use of perceptual access reasoning because the irrelevant option is one that the protagonist in the narrative is ignorant of or lacking perceptual access to. According to the PAR view, children who lack a fully developed ToM should choose between the belief and irrelevant options but avoid choosing the reality option.

To explore this, we examined children’s choices for each option on the modified False Belief tasks. Table 4 shows the number of children who chose each option for both Location and Contents versions of the modified 3- and 4-option False Belief tasks. Descriptively, both 4- and 5-year-olds showed low rates of choosing the reality option (Range: 7–41%), although it should be noted that choice of the reality option was higher on Contents versions of the tasks compared to Location versions among 4-year-olds (*p*s < 0.01). This task effect replicates prior work showing that 4-year-olds are more likely to choose the reality option on Contents versions of modified tasks compared to Location versions (Fabricius and Khalil, 2003; Fabricius et al., under review).

**TABLE 4:** Percent (Number) of 4- and 5-year-olds choosing each option by task type and version.

	3-option false belief			4-option false belief		
	Belief	Irrelevant	Reality	Belief	Irrelevant	Reality
**4-year-olds**						
Location	0.29 (12)	0.63 (26)	0.07 (3)	0.23 (9)	0.68 (26)	0.07 (3)
Contents	0.44 (19)	0.13 (6)	0.41 (18)	0.50 (20)	0.17 (7)	0.32 (13)
**5-year-olds**						
Location	0.57 (24)	0.33 (14)	0.09 (4)	0.52 (21)	0.42 (17)	0.05 (2)
Contents	0.78 (33)	0.04 (2)	0.16 (7)	0.72 (29)	0.10 (4)	0.17 (7)

Looking only at children who avoided the reality option, selection of the belief or irrelevant options varied. Binomial tests were conducted to test whether children’s choice of the belief option (when they avoided the irrelevant option) was greater than chance. Among 4-year-olds, rates of choosing the belief option across *Location* versions of the modified tasks were significantly below chance (*p*s < 0.05), whereas choice of the belief option was significantly higher than chance on *Contents* versions of the modified tasks (*p*s < 0.01). For 5-year-olds, selection of the belief option did not differ from chance on *Location* versions of the tasks, but was significantly above chance on *Contents* versions of the tasks (*p*s < 0.001). Thus, for children who avoided the reality option, there was greater selection of the irrelevant option on *Location* versions of the modified 3- and 4-option False Belief tasks, whereas they were generally correct in their selection of the belief option on *Contents* versions of the modified 3- and 4-option False Belief tasks.

### Relation Between Working Memory and Theory of Mind

Although these findings offer initial support in favor of a conceptual limitation account, driven by children’s performance on Location versions of the modified tasks, an alternative possibility is that modifying false belief tasks to include additional options might tax children’s working memory. Indeed, at test, children are tasked with reconstructing the sequence of events to correctly recall which location or object the protagonist has a false belief about. Thus, we examined contributions of working memory to children’s performance on False Belief tasks, which would lend support to executive accounts of ToM.

First, as shown in Table 2, we found significant correlations between the Working Memory Composite and performance on the modified 3-option False Belief Contents *r*(85) = 0.33, *p* < 0.01, and 3-option False Belief Location task *r*(85) = 0.28, *p* < 0.05. The Working Memory Composite was also correlated with the 4-option False Belief Contents *r*(85) = 0.24, *p* < 0.05 but not the 4-option False Belief Locations task. Working memory was not correlated with performance on True Belief or the standard 2-option False Belief tasks. When controlling for age, however, only the relation between Working Memory and the 3-option modified Contents False Belief task remained marginally significant (*r* = 0.19, *p* < 0.10).

Next, we examined whether working memory would predict success on the false belief tasks using logistic regression. As in the above analyses, for the modified tasks, we collapsed the two incorrect responses (irrelevant and reality) into one response category, yielding a score of 1 (belief) or 0 (irrelevant or reality). A logistic mixed effects model was conducted to predict score (1: Pass, 0: Fail) from the fixed effects of task order, age (months), task type (2-, 3, or 4-option False Belief), task version (Contents vs. Location), Working Memory Composite score, the interaction between task type and Working Memory, and the random effects (intercept) of participants. The analysis revealed significant main effects of task order, age, task type, and task version (see Table 5). Consistent with the analysis above, performance increased with age, was lower on modified 3- and 4-option versions of the False Belief task compared to the 2-option standard False Belief task, and was lower on Location versions of the modified False Belief tasks compared to Contents versions. Working memory was not related to performance.

**TABLE 5:** Results of mixed logistic regression model predicting odds of choosing belief by age, task order, task type, task version, and WM Composite.

Predictor	Odds ratios	Conf. int (95%)	*P*-value
Intercept	0.02	0.00–1.47	0.075
Task order	0.62	0.41–0.96	0.030
Age (months)	1.15	1.07–1.23	**<0.001**
Task (modified 3-options FB)	0.08	0.03–0.17	**<0.001**
Task (modified 4-option FB)	0.06	0.03–0.13	**<0.001**
Task version (location)	0.34	0.19–0.59	**<0.001**
WM composite	0.64	0.25–1.60	0.336
Task (modified 3-option FB) * WM composite	2.24	0.84–5.98	0.109
Task (modified 4-option FB) * WM composite	1.52	0.58–3.96	0.394
**Random effects**			
∫^2^	3.29		
∣00 subject	1.85		
ICC	0.36		
*N* subject	69		
Observations	414		
Marginal *R*^2^/Conditional *R*^2^	0.373/0.599		

*The reference group for Task was 2-option FB. The reference group for Task Version was Contents. Bold values are statistically significant values.*

This was followed up with ordinal logistic regression analysis (OLR) to preserve the three ordered response categories (belief, irrelevant, and reality). Separate OLRs were run for each version of the modified 3- and 4-option False Belief tasks. In all models, response was predicted by task order, age (months), and Working Memory Composite score. The results are shown in Tables 6, 7. The analyses revealed significant contributions of age to performance across both Location and Contents versions of the modified 3-option False Belief tasks (*ps* < 0.05) as well as on the Location version of the modified 4-option False Belief task (*p* < 0.01). The Working Memory Composite score was associated with performance on the Contents version of the 3-option False Belief task (*p* < 0.05). Task order was not associated with performance.

**TABLE 6:** Ordinal logistic regression models for 3-option false belief tasks.

				95% CI for *OR*	
Variable	б (*SE*)	*Z*	Odds ratio	Lower	Upper
**Location version**					
Task order	−0.37 (0.21)	−1.76	0.68	0.44	1.03
Age (months)	0.08 (0.03)*	0.03	1.09	1.02	1.17
WM composite	0.18 (0.29)	0.29	1.20	0.67	2.18
**Contents version**					
Task order	−0.25 (0.22)	−1.15	0.77	0.50	1.19
Age (months)	0.09 (0.03)*	2.40	1.09	1.01	1.19
WM composite	0.70 (0.32)*	0.32	2.02	1.09	3.87

CI, confidence interval; OR, odds ratio. *p < 0.05.

**TABLE 7:** Ordinal logistic regression models for 4-option false belief tasks.

				95% CI for *OR*	
Variable	б (*SE*)	*Z*	Odds ratio	Lower	Upper
**Location version**					
Task order	−0.24 (0.23)	−1.04	0.78	0.49	1.22
Age (months)	0.10 (0.03)**	2.63	1.10	1.02	1.19
WM composite	−0.07 (0.31)	−0.24	0.92	0.49	1.71
**Contents version**					
Task order	−0.21 (0.21)	−1.03	0.80	0.52	1.21
Age (months)	0.06 (0.03)	1.77	1.06	0.99	1.15
WM composite	0.47 (0.31)	1.48	1.60	0.86	3.02

CI, confidence interval; OR, odds ratio. **p < 0.01.

## Discussion

In light of recent work suggesting that preschool-aged children might lack a representational ToM, this study sought to determine how the addition of irrelevant response options influences performance, and whether individual differences in working memory relate to 4- and 5-year-olds’ performance on the modified False Belief tasks. In line with previous research, preschoolers performed worse on modified 3- and 4-option False Belief tasks and True Belief tasks compared to standard (2-option) False Belief tasks. We found that working memory was related to performance on the 3-option Contents but not Location version, and that age was the strongest predictor of passing modified False Belief tasks and True Belief tasks. These findings suggest that conceptual and executive limitations may play a role in the development of ToM.

### Performance on Modified False Belief Tasks

In the current study, preschoolers performed worse on modified False Belief tasks compared to standard False Belief tasks. This finding replicates and offers important extensions to previous reports. First, the pattern of responses found here are consistent with findings reported in a study by Fabricius and Khalil (2003). More specifically, children’s responses on modified False Belief tasks were largely constrained to the belief and irrelevant options, suggesting that their selections were not arbitrary. Fabricius and Khalil (2003) argue that children who use PAR attribute ignorance to agents and with two “wrong” options in the narrative (belief and irrelevant), thus selection of the two choices should fluctuate. In addition to task performance, we replicate an anomalous task version effect reported in two prior studies (Fabricius and Khalil, 2003; Perner and Horn, 2003) in which choice of the reality option was higher on Contents versions of the tasks compared to Location versions. According to the PAR hypothesis, preschoolers perform worse on Contents tasks because it is difficult to think of a “wrong” option when the options are not perceptually salient. Here, we found this pattern on both 3- and 4-option modified False Belief tasks for children in our 4-year-old age group. However, despite greater selection of the reality option amongst 4-year-olds on the Contents tasks, both 4- and 5-year-olds performed better (i.e., were more likely to provide the belief response) on Contents versions of the tasks compared to Location versions. This may be due to the use of boxes with familiar items depicted on the cover (Band-Aids and Cookies), serving as a salient reminder of the belief option when asked the test question on Contents tasks.

Second, our findings extend beyond the previous reports by including a 4-option False Belief task. To our knowledge, this is the first study to test for the contribution of adding more options to standard (2-option) False Belief tasks in a linear fashion. Although modified 3- and 4-option False Belief tasks were more difficult compared to standard 2-option False Belief tasks, the modified tasks did not differ from each other in difficulty. Control question performance was excellent across both 3- and 4-option versions of the tasks, suggesting that children accurately recalled the sequence of movements of the objects/locations described in the narrative. Moreover, children were not more likely to arbitrarily select among the four options in the 4-option tasks compared to the 3-option tasks, offering additional support that they may have utilized a reasoning strategy like PAR. Finally, we found the same task effect in the 4-option tasks, such that there were higher rates of selecting the reality option on Contents compared to Location, but that selection of the belief response (among those who avoided reality) was higher. Again, this may be attributed to the fact that the familiar contents on the box served as a reminder of the belief option when asked the test question. Future work could explore for differences in task version by asking the test question without the box present at test. Given the similar pattern of responses across the set of modified tasks administered here, it is plausible that a similar cognitive process is functioning on false belief tasks regardless of the number of options.

### Contributions of Working Memory to False Belief Performance

One possible explanation, which we investigated here, was that the addition of objects/locations to the narrative would impose greater executive demands. Whereas previous work argues that conceptual limitations, driven by preschoolers’ use of PAR, account for their performance on modified 3-option False Belief tasks, we investigated this from a different lens. Specifically, we focused on how the neurocognitive processes involved in executive function might explain performance on modified False Belief tasks. Due to an expectation that working memory would be particularly taxed by the demands of the modified False Belief tasks, we focused on this component of executive function in the present work.

We found weak associations between working memory and children’s performance on modified False Belief tasks. After controlling for age, significant positive correlations between working memory and performance on modified 3- and 4-option False Belief tasks disappeared. This also held for standard 2-option False Belief tasks. Further, ordinal logistic regression analyses found that the Working Memory Composite score was only associated with performance on the modified 3-option Contents False Belief task. This suggests that working memory alone cannot explain performance on False Belief tasks, extending to both task type (2-, 3-, and 4-option) and task version (Contents and Location). These results were unexpected given previous reports on the relation between working memory and false belief performance (Devine and Hughes, 2014). Despite this, it is important to note that we did not include measures of inhibitory control or cognitive flexibility, both of which have been found to relate to performance on standard (2-option) False Belief tasks (Carlson et al., 2002). Thus, it remains possible that the neurocognitive processes involving executive function contribute to children’s false belief understanding.

### True Belief Performance

We included True Belief tasks in response to work reporting poor performance on these tasks at this age (Fabricius et al., 2010). Like others (Oktay-Gür and Rakoczy, 2017; Rakoczy and Oktay-Gür, 2020), we found that children performed worse on True Belief tasks compared to standard 2-option False Belief tasks. Specifically, whereas 5-year-olds selected the belief option at above chance levels, 4-year-olds were at chance on both Contents and Location versions despite performing well on standard False Belief tasks. This pattern has been argued to support the PAR hypothesis; that is, children at this age who reason that an agent who does not have current perceptual access will “get it wrong” should provide the belief response on standard 2-option False Belief tasks and the reality response on 2-option True Belief tasks (Hedger and Fabricius, 2011). Another explanation, recently offered by Rakoczy and Oktay-Gür, suggests that poor performance on True Belief tasks is due to the confusing pragmatics involved, which can be remedied by administering True Belief before False Belief or by changing the test question wording. Yet another possibility is that True Belief tasks impose executive demands we have failed to take into consideration. Indeed, research on EF and ToM has focused on standard False Belief task performance, leaving uninvestigated the role for EF on reasoning about false beliefs *and* true beliefs. Though we failed to find evidence of a positive relationship between working memory and true belief performance in the present study, it remains possible that other components of EF may play a role in true belief reasoning.

### Conceptual, Pragmatic, and Executive Limitations

Our results speak to growing debate in the literature surrounding children’s ToM development. The disparate performance on these tasks suggests to some scholars conceptual limitations to children’s belief understanding, whereas others point to pragmatic limitations masking children’s conceptual abilities.

On the PAR hypothesis, conceptual limitations are evident in children’s attributions of ignorance to agents. Our results offer support for this view, specifically among 4-year-olds. As predicted by the PAR hypothesis, 4-year-olds performed worse on modified 3- and 4-option False Belief tasks compared to 2-option False Belief tasks. Specifically, their pattern of responding aligns with the PAR prediction that there should be few selections of the reality response and more equal selection of the belief and irrelevant responses. In addition, 4-year-olds’ performance across standard 2-option False Belief tasks and True Belief tasks supports the PAR prediction that children who are reasoning about ignorance should pass standard False Belief tasks but fail True Belief tasks. Previous investigations of the PAR hypothesis have demonstrated these predicted patterns of responses, but used these tasks across different studies, administering modified False Belief tasks (Fabricius and Khalil, 2003) or standard 2-option False Belief vs. True Belief (Fabricius et al., 2010). The current study offers a unique contribution to this body of evidence by administering all of these tasks in the same within-participant design, and finding the predicted pattern across the battery of ToM tasks. If conceptual limitations are responsible for children’s pattern of responding, we would expect to find that a reasoning strategy (like PAR) functions across a set of tasks for children using that heuristic, which we report here. This suggests that 4-year-olds may lack a fully representational ToM despite passing the standard False Belief tasks.

These findings offer minimal support for a pragmatic limitation account. Support for a pragmatics account is drawn from findings that children can pass modified True Belief tasks, for example if characters are present for the critical location change (Oktay-Gür and Rakoczy, 2017) or if children are told questions might be trivial because they are intended for younger children (Rakoczy and Oktay-Gür, 2020). While the present study did not systematically test for pragmatic differences across our ToM battery, recent work has found that manipulating various aspects of modified 3-option False Belief tasks (e.g., manipulations to the narrative, movements of the object, which movements the protagonist witnessed) did not affect performance in any predictable fashion (Fabricius et al., under review). Thus, to date, there is scant evidence supporting the predictions made by pragmatic accounts, suggesting there may be a deeper reason for children’s failures.

In a large literature, facets of EF have been shown to relate to children’s ToM development, leading scientists to wonder whether EF allows for the conceptual understanding involved in belief reasoning (emergence accounts) or is a constraint on the expression of an already-developed conceptual understanding of belief (expression accounts) (Moses, 2001). While this debate was not at the center of the work conducted here, our findings suggest that EF might play a role in performance on modified False Belief (specifically, 3-option Contents False Belief), though it remains unclear whether this relation supports an emergence or expression account. On the one hand, although we had predicted that additional options would tax working memory, children’s working memory scores were not a strong predictor of passing modified 3- and 4-option False Belief tasks. This suggests that the working memory demands of the additional task events were minimal. On the other hand, it may be that EF plays a role in the conceptual understanding involved in reasoning on modified tasks, in ways that we failed to test for here. More robust exploration of the relation between EF (including working memory, inhibitory control, and cognitive flexibility) has the potential to reveal more about our understanding of ToM development in the preschool years. For instance, finding that EF relates to performance on more than standard False Belief tasks would suggest that EF is critical to belief reasoning across a range of tasks.

### Limitations

This study has a number of limitations. First, as just discussed, while we expected working memory to be particularly taxed by the addition of irrelevant options on modified False Belief tasks, it might have been prudent to also assess other components of EF. This would have allowed us to better determine the impact that specific components of EF have on performance across modified False Belief tasks (as well as True Belief tasks, though this was not the primary aim of the current study). Thus, in future work, it will be important to include a larger EF battery to examine the contributions of EF on ToM. Second, while we situate our results within the debate surrounding conceptual, pragmatic, and executive demands, we did not systematically manipulate pragmatics of the tasks. To this end, we cannot rule out the possibility that pragmatic demands might have accounted for our findings, in ways recently proposed by Oktay-Gür and Rakoczy (2017) and Rakoczy and Oktay-Gür (2020). Third, our sample was predominantly White and from high socioeconomic households, limiting our ability to generalize these findings and conclusions to other populations.

## Conclusion

Although standard False Belief tasks have acquired tenure as a marker of ToM understanding, recent empirical work suggests there may be limitations to preschoolers’ understanding of belief. The work presented here is one of the first to take into consideration varied accounts of ToM development by administering standard and modified False Belief tasks, True Belief tasks, and measures of working memory in the same within-participant design. We sought to determine whether previous reports using these tasks would replicate when administered in the same testing session, including the addition of 4-option task versions, and whether individual differences in working memory would explain children’s performance on this unique ToM battery. Our findings speak to a rising debate within the literature about how to interpret children’s responses to different types of ToM tasks. We found evidence in favor of a conceptual limitation account, given children’s poor performance on both modified False Belief tasks and True Belief tasks (despite their strong performance on standard 2-option False Belief tasks). In addition, we found preliminary evidence in favor of an executive account given the association between working memory and performance on the modified 3-option Contents False Belief task. Finally, we found little evidence supporting a pragmatics account given the lack of an association between task order and task performance. While we cannot settle the debate here, the growing body of work examining preschoolers’ performance on different ToM tasks suggests that more research is needed to better understand preschoolers’ development of belief reasoning. We add to the current debate the possibility that executive demands play a role in children’s performance on these modified tasks in addition to the possibility of conceptual and pragmatic limitations. It is important to consider the implications of these findings, given the robust use of standard False Belief tasks to assess ToM in developmental research.

## Data Availability Statement

The raw data supporting the conclusions of this article will be made available by the authors, without undue reservation.

## Ethics Statement

The studies involving human participants were reviewed and approved by Institutional Review Board at the University of Minnesota. Written informed consent to participate in this study was provided by the participants’ legal guardian/next of kin.

## Author Contributions

AS, AP, and SC conceptualized the study. AS and AP collected, analyzed, and interpreted the data. AS and AP drafted the manuscript. SC critically reviewed the manuscript. All authors gave their final approval of the manuscript.

## Conflict of Interest

The authors declare that the research was conducted in the absence of any commercial or financial relationships that could be construed as a potential conflict of interest.

## References

[B1] Carlson S. M. Koenig M. A. Harms M. B. (2013). Theory of mind. *Wiley Interdiscip. Rev. Cogn. Sci.* 4 391–402. 10.1002/wcs.1232 26304226

[B2] Carlson S. M. Moses L. J. (2001). Individual differences in inhibitory control and children’s theory of mind. *Child Dev.* 72 1032–1053. 10.1111/1467-8624.00333 11480933

[B3] Carlson S. M. Moses L. J. Breton C. (2002). How specific is the relation between executive function and theory of mind? Contributions of inhibitory control and working memory. *Infant Child Dev.* 11 73–92. 10.1002/icd.298

[B4] Carlson S. M. Moses L. J. Hix H. R. (1998). The role of inhibitory processes in young children’s difficulties with deception and false belief. *Child Dev.* 69 672–691. 10.1111/j.1467-8624.1998.tb06236.x 9680679

[B5] Clements W. A. Perner J. (1994). Implicit understanding of belief. *Cogn. Dev.* 9 377–395. 10.1016/0885-2014(94)90012-4

[B6] Corsi P. M. (1972). *Human Memory and the Medial Temporal Region of the Brain*, Vol. 34 Dissertation Abstracts International, University Microfilms, Ann Arbor, MI, 819B.

[B7] Devine R. T. Hughes C. (2014). Relations between false belief understanding and executive function in early childhood: a meta-analysis. *Child Dev.* 85 1777–1794. 10.1111/cdev.12237 24605760

[B8] Fabricius W. V. Boyer T. W. Weimer A. A. Carroll K. (2010). True or false: do 5-year-olds understand belief? *Dev. Psychol.* 46 1402–1416. 10.1037/a0017648 21058830

[B9] Fabricius W. V. Khalil S. L. (2003). False beliefs or false positives? Limits on children’s understanding of mental representation. *J. Cogn. Dev.* 4 239–262. 10.1207/S15327647JCD0403_01

[B10] Gordon A. C. Olson D. R. (1998). The relation between acquisition of a theory of mind and the capacity to hold in mind. *J. Exp. Child Psychol.* 68 70–83. 10.1006/jecp.1997.2423 9473316

[B11] Happé F. G. (1994). An advanced test of theory of mind: understanding of story characters’ thoughts and feelings by able autistic, mentally handicapped, and normal children and adults. *J. Autism. Dev. Disord.* 24 129–154. 10.1007/BF02172093 8040158

[B12] Hedger J. A. Fabricius W. V. (2011). True belief belies false belief: recent findings of competence in infants and limitations in 5-year-olds, and implications for theory of mind development. *Rev. Philos. Psychol.* 2 429–447. 10.1007/s13164-011-0069-9

[B13] Hogrefe G. J. Wimmer H. Perner J. (1986). Ignorance versus false belief: a developmental lag in attributing epistemic states. *Child Dev.* 57 567–582. 10.2307/1130337

[B14] Miller S. A. (2009). Children’s understanding of second-order mental states. *Psychol. Bull.* 135 749–773. 10.1037/a0016854 19702381

[B15] Miyake A. Friedman N. P. Emerson M. J. Witzki A. H. Howerter A. Wager T. D. (2000). The unity and diversity of executive functions and their contributions to complex “Frontal Lobe” tasks: a latent variable analysis. *Cogn. Psychol.* 41 49–100. 10.1006/cogp.1999.0734 10945922

[B16] Moses L. J. (2001). Executive accounts of theory of mind development. *Child Dev.* 72 688–690. 10.1111/1467-8624.00306 11405573

[B17] Oktay-Gür N. Rakoczy H. (2017). Children’s difficulty with true belief tasks: competence deficit or performance problem? *Cognition* 166 28–41. 10.1016/j.cognition.2017.05.002 28554083

[B18] Perner J. (1991). *Understanding the Representational Mind.* Cambridge, MA: MIT Press.

[B19] Perner J. Horn R. (2003). Ignorance or false negatives: do children of 4 to 5 years simulate belief with “not knowing = Getting it wrong?”. *J. Cogn. Dev.* 4 263–273. 10.1207/S15327647JCD0403_02

[B20] Rakoczy H. Oktay-Gür N. (2020). Why do young children look so smart and older children look so dumb on true belief control tasks? An investigation of pragmatic performance factors. *J. Cogn. Dev.* 21 213–239. 10.1080/15248372.2019.1709467

[B21] Wellman H. M. Cross D. Watson J. (2001). Meta-analysis of theory-of-mind development: the truth about false belief. *Child Dev.* 72 655–684. 10.1111/1467-8624.00304 11405571

[B22] Wellman H. M. Liu D. (2004). Scaling of theory-of-mind tasks. *Child Dev.* 75 523–541. 10.1111/j.1467-8624.2004.00691.x 15056204

[B23] Wimmer H. Perner J. (1983). Beliefs about beliefs - representation and constraining function of wrong beliefs in young children’s understanding of deception. *Cognition* 13 103–128. 10.1016/0010-0277(83)90004-5 6681741

[B24] Zelazo P. D. Blair C. B. Willoughby M. T. (2016). *Executive Function: Implications for Education.* Washington, DC: National Center for Education Research.

